# Testing Small-Strain Dynamic Characteristics of Expanded Polystyrene Lightweight Soil: Reforming the Teaching of Engineering Detection Experiments

**DOI:** 10.3390/polym17060730

**Published:** 2025-03-10

**Authors:** Ping Jiang, Xinghan Wu, Lejie Chen, Na Li, Erlu Wu

**Affiliations:** Shaoxing Key Laboratory of Interaction Between Soft Soil Foundation and Building Structure, School of Civil Engineering, Shaoxing University, Shaoxing 312000, China; jiangping@usx.edu.cn (P.J.); 23020859210@usx.edu.cn (X.W.); 21020859007@usx.edu.cn (L.C.); lina@usx.edu.cn (N.L.)

**Keywords:** EPS lightweight soil, small strain, resonant column test, dynamic shear modulus, damage model, engineering detection experiment, teaching reform

## Abstract

This study investigated the small-strain dynamic properties of expanded polystyrene (EPS) lightweight soil (ELS), a low-density geosynthetic material used to stabilize slopes and alleviate the subgrade settlement of soft soil. Resonant column tests were conducted to evaluate the effects of EPS’s granule content (20–60%), confining pressures (50 kPa, 100 kPa, and 200 kPa), and curing ages (3 days, 7 days, and 28 days) on the dynamic shear modulus (*G*) of ELS within a small strain range (10^−6^–10^−4^). The results indicate that ELS exhibits a high dynamic shear modulus under small strains, which increases with higher confining pressure and longer curing age but decreases with an increasing EPS granule content and dynamic shear strain, leading to mechanical property deterioration and structural degradation. The maximum shear modulus (*G*_max_) ranges from 64 MPa to 280 MPa, with a 60% reduction in *G*_max_ observed as the EPS granule content increases and increases by 11% and 55% with higher confining pressure and longer curing ages, respectively. A damage model incorporating the EPS granule content (*a*_E_) and confining pressure (*P*) was established, effectively describing the attenuation behavior of *G* in ELS under small strains with higher accuracy than the Hardin–Drnevich model. This study also developed an engineering testing experiment that integrates materials science, soil mechanics, and environmental protection principles, enhancing students’ interdisciplinary knowledge, innovation, and practical skills with implications for engineering construction, environmental protection, and experimental education.

## 1. Introduction

Expanded polystyrene (EPS), a type of expanded plastic material made from steam-expanded polystyrene beads, is commonly used in construction and insulation applications due to its lightweight and durable nature. When EPS particles are incorporated into lightweight soil, they contribute to the creation of expanded polystyrene lightweight soil (ELS), a novel geotechnical material characterized by its lightweight, high strength, durability, and excellent self-supporting properties [1]. ELS demonstrates promising application prospects in addressing geotechnical and environmental issues, with its feasibility demonstrated in soft soil foundation treatment, backfill behind retaining walls, and roof insulation engineering [2,3]. The uniformity of the EPS particle distribution within the soil plays a crucial role in enhancing the bearing capacity of soft soil foundations [4]. Additionally, the resource utilization of polystyrene foam, such as combining recycled EPS waste with mortar to form lightweight bricks, effectively reduces white pollution [5,6]. In the fields of foundation, slope, and geotechnical engineering, most soil structures are often in a small strain state (10^−6^ to 10^−4^), and the small deformations of soil under dynamic loads have garnered significant attention [7,8,9]. However, there is a notable discrepancy between the soil stiffness at small strain levels and that obtained through traditional tests, highlighting the importance of considering small-strain characteristics when studying soil deformation and stiffness. Furthermore, accurately assessing the small-strain shear modulus is essential for analyzing the deformation behavior and dynamic performance of ELS under dynamic loading conditions [10,11,12].

Currently, several trial studies have been conducted to explore the dynamic shear modulus of ELS at small strains, employing methods such as resonant column tests, bending element tests, and dynamic triaxial tests. Sherbiny et al. [13], using resonant column tests, investigated the dynamic properties of light soil combined with EPS–sand particles at different confining pressures and minor strains. It was discovered that a 0.5% increase in the number of EPS particles led to a 12% reduction in soil weight, which was associated with a drop in shear stiffness. To investigate the stiffness characteristics of expanded polystyrene composite soil under small strains, Gao et al. [14] carried out unconfined compressive tests and resonant column trials. The findings showed that the unconfined compressive strength and shear modulus of the expanded polystyrene composite soil increased with rising cement and confining pressure levels and declined with the increasing EPS particle content. Using cyclic triaxial and bending element experiments, Alaie et al. [15] investigated how the small-strain shear modulus of lightweight soil containing a mixture of sand and EPS particles was affected by the amount of EPS particle content, confining pressure, and shear-strain amplitude. The findings demonstrate that, although the dynamic shear modulus obtained from the cyclic triaxial test was largely reliant on the EPS particle content, as the EPS particle admixture increased, the shear wave velocity and shear modulus magnitude measured using the bending element test dropped. Bekranbehesht et al. [16] used bending element testing to examine the impact of EPS particles and confining pressure on the mixed quartz and calcareous sands’ small-strain dynamic shear modulus. For the mixture of both sands, the maximum dynamic shear modulus was found to decrease with an increase in EPS particle mixing and rise with an increase in confining pressure. In addition, Gao et al. [17] examined the dynamic shear modulus of EPS-blended soils and discovered that EPS particle doping had no discernible effect on blended soils’ initial dynamic shear modulus as a result of the strength of the cemented structure and the initial stress level. However, the blended soil’s dynamic shear modulus was significantly weakened by the EPS particles when the soil entered the nonlinear phase. Zhu et al. [18] performed dynamic triaxial testing on a blend of lightweight soil, sand, and EPS particles. Their analysis focused on how confining pressure, the EPS particle mixture, cement content, and cycle count affected the dynamic shear modulus attenuation of the lightweight soil. Additionally, they developed a model to predict the dynamic shear modulus’ attenuation. In summary, the majority of academic studies primarily examine how the doping of EPS particles and confining pressure affect lightweight soil’s dynamic shear modulus. However, as soil engineering performance standards and structural safety and stability are progressively improved, it is also critical to investigate how soil’s dynamic shear modulus is affected by curing age [19]. At the same time, most researchers have underexplored the decay law and damage mechanism of the small-strain shear modulus (*G*) of ELS. Furthermore, there is a lack of mathematical models describing the relationship between confining pressure, EPS particle content, and the dynamic shear modulus of ELS.

The effect of different EPS particle contents, confining pressures, and curing ages on the small-strain *G* of ELS was investigated using resonant column tests. Due to the complex composition and structure of ELS, its porosity and structural properties show obvious differences under different mixing ratios, leading to the damage characteristics of the specimens under dynamic loading conditions. By adding damage variables, a small-strain damage model was created based on damage theory and the experimental findings, which were used to characterize the attenuation of the dynamic shear modulus of ELS specimens with the increase in dynamic shear strain. For the application of ELS in foundation treatment, highways, and bridges, theoretical underpinnings and data references are provided.

At present, traditional undergraduate experiments such as soil mechanic experiments, detection experiments, physics experiments, and structural experiments are closely related to theoretical courses in civil engineering majors [20]. However, these independent experimental projects lack corresponding internal connections, which cannot meet the needs of civil engineering research and development and also limit the cultivation of students’ innovative thinking. Therefore, it is urgent to reform the teaching of engineering detection experiments for undergraduate students. In order to address the impact of EPS on the environment and explore the mechanical properties of ELS under small-strain dynamic loads while further improving students’ engineering testing and experimental skills, broadening their knowledge, and enhancing their innovation ability, this paper details the design of an innovative engineering detection experimental project that integrates environmental science, materials science, and soil mechanics. The influence of different EPS particle contents, confining pressures, and curing ages on the dynamic shear modulus of ELS was studied through resonant column tests. This experimental project can not only help students integrate existing fragmented knowledge and solve scientific research problems but also enable them to master experimental skills in the fields of environmental science, materials science, and soil mechanics, thereby enhancing their innovation ability.

## 2. Materials and Methods

### 2.1. Materials

**Figure 1:**
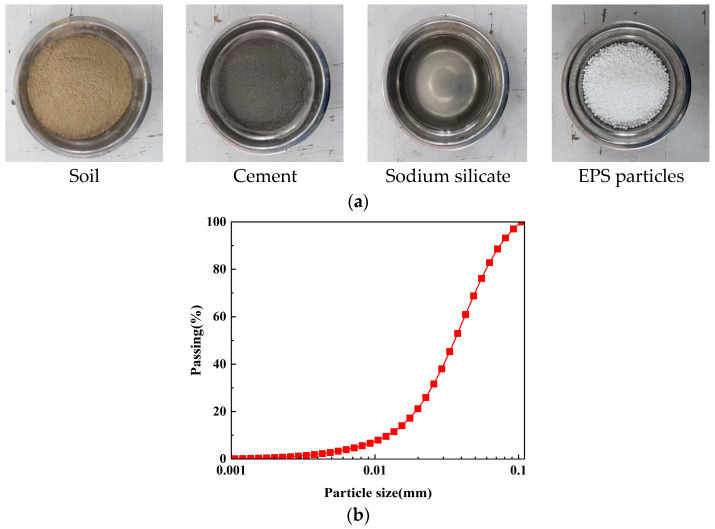
Materials: (**a**) material composition of ELS; (**b**) soil particle gradation chart.

ELS is composed of soil, EPS particles, cement, a sodium silicate solution, and water, with the raw materials shown in Figure 1a. The soil used in the experiment was collected from a renovation project site in Shaoxing City, Zhejiang Province, and it exhibited a yellowish-brown color. The basic physical properties of the soil were tested according to the Test Methods of Soils for Highway Engineering (JTG 3430-2020) [21], and the results are presented in Table 1. After being dried and crushed, the soil was subjected to particle size analysis, revealing that it primarily consists of sand particles, as illustrated in Figure 1b. Based on preliminary experimental preparations, to achieve the goal of lightweight design and high strength in the ELS specimens, EPS particles with a diameter of 2–3 mm were selected. These particles had a bulk density of 0.018 g/cm^3^, pure particle density of 0.0314 g/cm^3^, and compressive strength of up to 170 kPa, demonstrating good durability. The curing agents used were cement and sodium silicate solution. Considering both strength and cost-effectiveness, the cement type chosen was P.C. 42.5 composite Portland cement, and the sodium silicate solution had a concentration of 40% and a modulus of 3.2. Ordinary tap water was used for the experiments. The diversity of experimental materials can enhance students’ interest in learning and stimulate their innovative thinking.

### 2.2. Specimen Preparation

**Figure 2:**
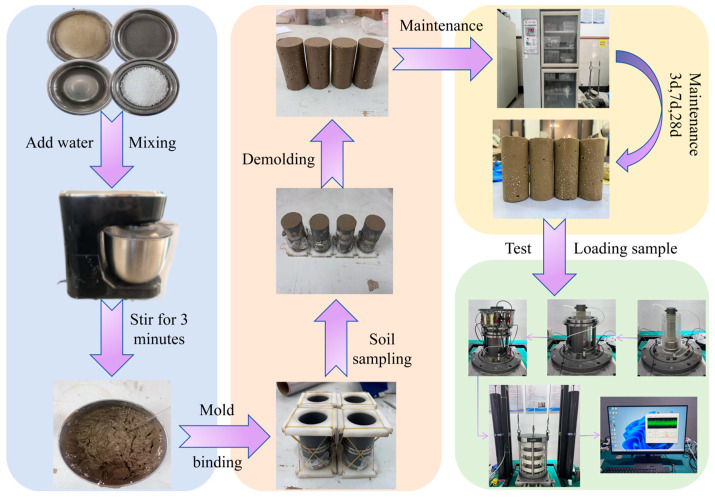
Specimen-making process.

According to the standard for geotechnical testing methods GB/T 50123-2019 [22], to minimize sample preparation errors, resonance column tests were conducted using 15 cylindrical specimens with a diameter of 50 mm and a height of 100 mm. The preparation process for the ELS specimens is given as follows: (1) Weigh the required masses of raw soil, EPS particles, cement, sodium silicate solution, and water according to the test mix ratio. (2) Thoroughly mix the soil, cement, sodium silicate solution, and water, and stir for 3 min. (3) Add the EPS particles and stir uniformly for another 3 min to ensure even dispersion of the EPS particles within the soil, forming a flowable ELS mixture. (4) Pour the prepared ELS mixture into cylindrical molds measuring 50 mm × 100 mm. Each mold is filled in three layers, with each layer undergoing vibration 10 times to ensure the mixture is dense and uniform. After pouring, cover the molds with plastic wrap and allow them to sit for 4 h for initial hardening. (5) Once the specimens have initially hardened, level and demold them to obtain standard cylindrical specimens with a diameter of 50 mm and a height of 100 mm. Wrap the specimens in plastic wrap and place them in a standard curing chamber, maintaining a temperature of 20 ± 2 °C and a relative humidity of 95% or higher. The specimen preparation process is illustrated in Figure 2.

### 2.3. Test Program

#### 2.3.1. Resonance Column Trialing System

**Figure 3:**
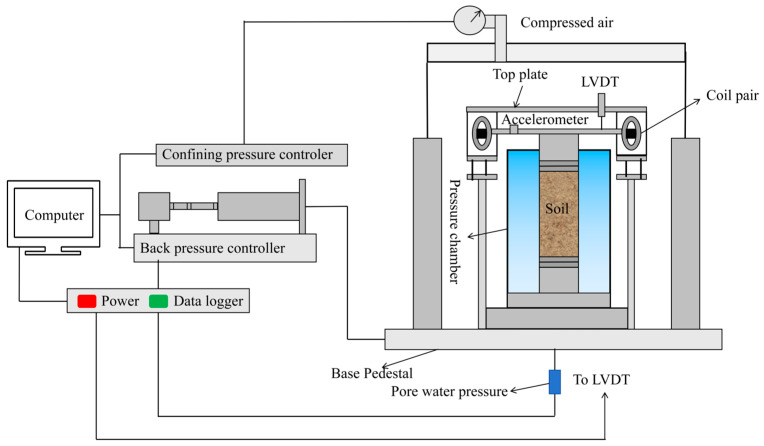
Resonant column instrument.

The resonant column test is used to determine the dynamic shear modulus of ELS at small strains (10^−6^–10^−4^). The test was conducted using the GDS-RCA resonant column system, which consists of a counterpressure controller, a data collection system, an electromagnetic drive system, a base, an accelerometer, and a computer. The test mode involves excitation through torsion. The structure of the instrument is shown in Figure 3.

#### 2.3.2. Resonance Column Test Program

To investigate the variation law of the dynamic shear modulus of ELS under small strains, resonant column tests were conducted on specimens with different EPS particle contents, confining pressures, and curing ages. To more accurately simulate the stress state of ELS at different soil depths, three different confining pressures were selected: 50 kPa, 100 kPa, and 200 kPa [23]. Table 2 presents the resonant column test scheme. The proportions of EPS particles, cement, sodium silicate, and water in the table are all percentages relative to the weight of dry soil.

#### 2.3.3. Resonance Column Test Procedure

The steps for the resonant column test are given as follows: (1) Place the ELS sample to be tested into a rubber membrane, secure it on the instrument base, and cover it with a top cap. Fix both ends with rubber rings. Then, install the waterproof cover and slowly inject pure water around the sample until it submerges the top of the sample. Connect the electromagnetic drive system to the top cap of the sample and level the equipment to ensure each magnet is centered within the coil. After leveling, install the accelerometer and drainpipe and lower the pressure chamber to complete the sample installation. (2) Open the GDS-RCA testing system and set the corresponding parameters according to the testing program, such as 50, 100, or 200 kPa confining pressure. After setting the parameters, select the “Torsion” test module in the system software. Apply a sinusoidal voltage of 0.01 V to the electromagnetic coil to generate torque on the sample and perform a wide-frequency scan on the acceleration signal from the accelerometer to obtain the frequency corresponding to the maximum voltage peak. (3) Based on the frequency range obtained from the wide-frequency scan, apply a voltage of 0.01–1 V to the electromagnetic coil during the fine scan to determine the sample’s resonant frequency. The measured resonant frequency range for the ELS sample is 38.3–108.2 Hz. (4) Calculate the sample’s moment of inertia based on its outer diameter (*d*) and mass (*m*). Determine the *β* value for different samples by referring to a table based on the ratio of the sample’s moment of inertia (*I*) to the resonant column drive system’s moment of inertia (*I*_0_). (5) Calculate the shear wave velocity (*V*_s_) using the resonant frequency (*f*), β value, and sample height (*H*). Finally, determine the dynamic shear modulus (*G*) of the sample based on the sample density (*ρ*) and shear wave velocity (*V*_s_). (6) By installing an accelerometer on the drive disk, the resonance output voltage and resonance frequency of the specimen are obtained. Based on the geometric dimensions of the specimen, the dynamic shear strain is calculated. The specific formulas are given as Equations (1)–(5):

(1) $$I=\frac{md^{2}}{8}$$

where *I* is the moment of inertia of the sample; *m* is the mass (kg); and *d* is the outside diameter (m).

(2) $$I/I_{0}=\beta \tan\left(\beta \right)$$

Here, *I*_0_ is the moment of inertia of the resonant column drive system (kg·m^2^).

(3) $$V_{s}=\frac{2\pi fH}{\beta }$$

Here, *V_s_* is the shear wave velocity (m/s); *f* is the resonance frequency (Hz); *H* is the sample height (m); and *β* is the eigenvalue of the torsional vibration equation (kg·m^2^).

(4) $$G=\rho \cdot V_{s}^{2}$$

Here, *G* is a dynamic shear modulus (MPa), and *ρ* is the sample density (t/m^3^).

(5) $$\gamma =\frac{4.596V\cdot d}{f^{2}\cdot H}$$

Here, *γ* is a dynamic shear strain (%), and *V* is the resonance output voltage (Volts).

## 3. Results and Analysis

### 3.1. Density and Shear Wave Velocity of ELS

Two important factors that affect the soil’s dynamic shear modulus are density and shear wave velocity [24,25].

**Figure 4:**
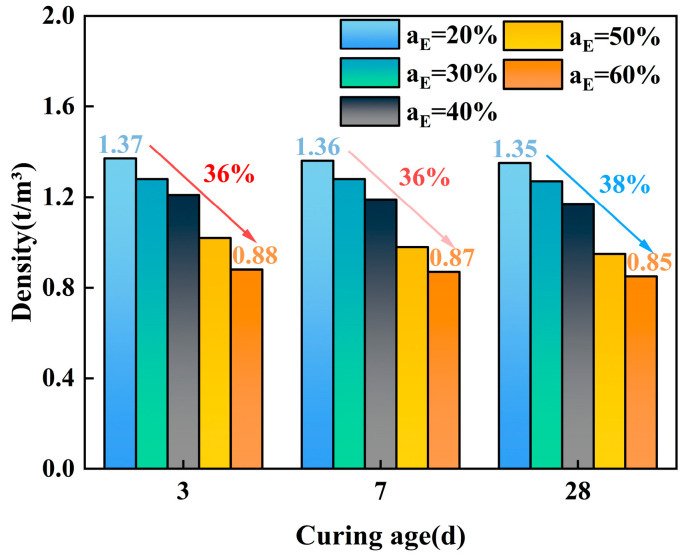
Density of ELS at different EPS particle contents and curing ages.

Figure 4 shows the variation in density for specimens with different EPS particle contents at different curing ages. It indicates that at the curing ages of 3 days, 7 days, and 28 days, as the EPS particle content increases, the density of ELS gradually decreases. As the curing age increases, the density of the specimens with a 20% to 60% EPS particle content decreases by 36%, 36%, and 38%, respectively. Generally, the density varies from 0.85 t/m^3^ to 1.37 t/m^3^.

**Figure 5:**
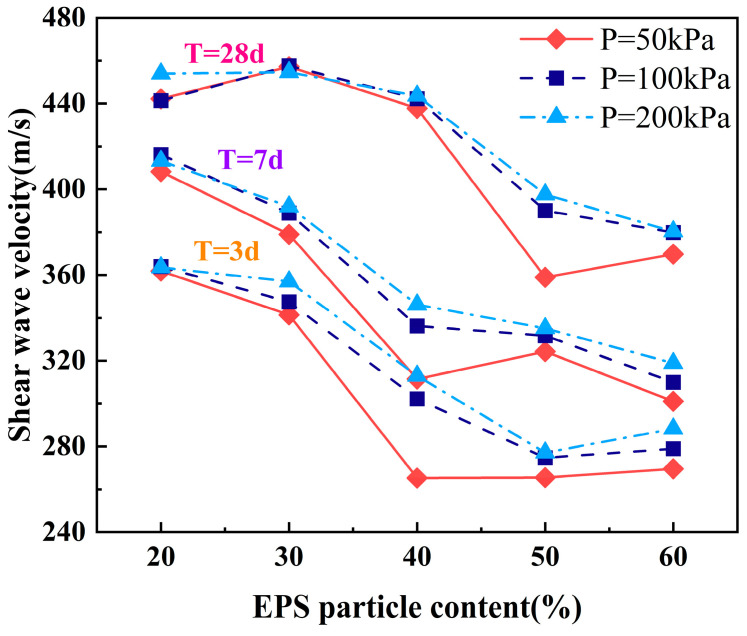
Shear wave velocity of ELS at different EPS particle contents, confining pressures, and curing ages.

Figure 5 illustrates the variation in shear wave velocity of ELS specimens under the influence of different EPS particle contents (a_E_), confining pressures, and curing ages. It shows that with an increase in the EPS particle content, the shear wave velocity of ELS generally decreases. Taking the 7-day curing age and 100 kPa confining pressure as an example, compared to the 20% EPS particle content, the shear wave velocities at the 30%, 40%, 50%, and 60% EPS particle contents decrease by 7%, 19%, 20%, and 26%, respectively. Additionally, using a 40% EPS particle content as a boundary, there is a significant decrease in shear wave velocity from low content (a_E_ = 20%, 30%) to high content (a_E_ = 50%, 60%). This decrease is related to the mechanism by which EPS particles affect the soil. As an expansive particle, the incorporation of EPS into the soil reduces the density of the specimen, leading to a looser internal structure of the soil and a decrease in shear wave velocity [26].

Under the same curing age, the shear wave velocity of ELS specimens with various EPS particle contents increases with the rise in confining pressure. The increase in shear wave velocity for specimens with higher EPS particle contents is significantly greater than that for those with lower contents. For example, at a 7-day curing age, the shear wave velocity of the specimen with a 20% particle content increases by 1% with an increase in the confining pressure, while the specimen with a 60% particle content shows an increase of 6%. This is mainly because the increase in confining pressure compresses the specimen’s pores, making the ELS specimens denser. The porosity of specimens with a high EPS particle content is larger than that of those with a low content, resulting in a more noticeable compression of the specimens with increasing confining pressure [27]. Meanwhile, as the curing age extends, the shear wave velocity of ELS specimens gradually increases, which is closely related to the hydration reaction of the material. In the early stages of curing, the reaction between the cement particles and water is not yet complete, leading to a relatively loose internal specimen structure and a lower shear wave velocity. As the curing time increases, the degree of cement hydration improves, forming more hydration products, which enhances the density and strength of the specimen and thereby increases the shear wave velocity [28].

### 3.2. Curves of Dynamic Shear Modulus G and Dynamic Shear Strain γ

The dynamic shear modulus is a crucial parameter for examining the stiffness properties of soil in numerical simulations, serving as a standard that indicates the material’s bearing capacity [29]. Dynamic shear modulus and dynamic shear strain (*G*–*γ*) relationship curves were established for varying EPS particle mixing amounts, confining pressures, and curing ages.

#### 3.2.1. Impact of Adding EPS Particles on the *G*–*γ* Relationship Curve

**Figure 6:**
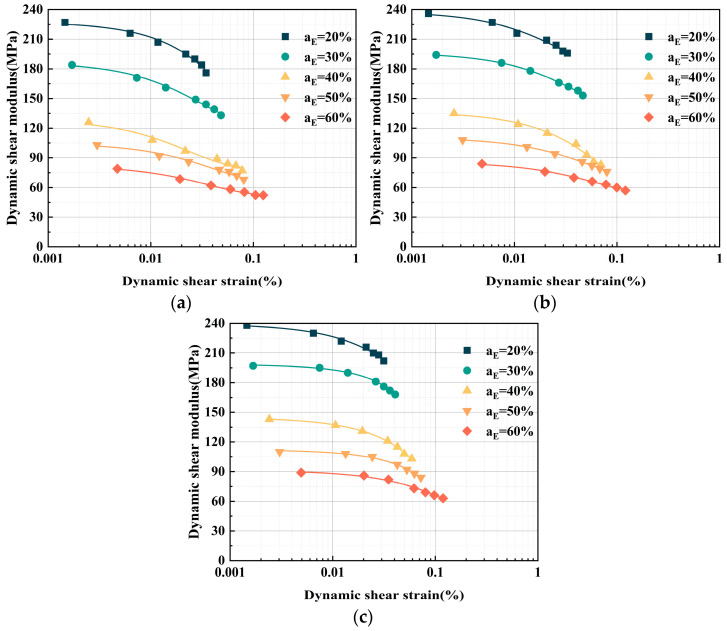
ELS *G*–*γ* relationship curves with different EPS particle contents. (**a**) Confining pressure = 50 kPa; (**b**) confining pressure = 100 kPa; and (**c**) confining pressure = 200 kPa.

When ELS is combined with EPS particles, the porosity, and compressibility of the specimen increase to some extent due to the low density of EPS particles and the porous structure between the particles. Additionally, during the cement hydration process, the hydration products (C-S-H) generated from the reaction between cement and water exhibit poor adhesion to the surface of EPS particles, making it difficult to fill the pores between the particles, which, in turn, affects the mechanical properties of the specimen [30]. The distribution of EPS particles also influences the uniformity of the specimen’s structure, further regulating the changes in porosity and compressibility [3]. Simultaneously, the addition of EPS particles leads to a decrease in the overall density of ELS. As clearly shown in Equation (4), the reduction in density results in a decrease in the dynamic shear modulus of the specimen. Taking the 7-day curing age data as an example, the influence of the EPS particle content on the *G*–*γ* relationship curve of ELS under confining pressures of 50 kPa, 100 kPa, and 200 kPa is illustrated in Figure 6.

As the dynamic shear strain (*γ*) increases, the dynamic shear modulus (*G*) of ELS exhibits a declining tendency at different EPS particle contents. In the initial stage (0.0015% < *γ* < 0.0073%), *G* decreases slowly. When *γ* > 0.01%, the rate of decline gradually accelerates, and the curve as a whole shows a characteristic nonlinear decline. At the same time, with the increase in the EPS particle content, *G* decreases, and the *G*–*γ* curve shifts to the right, meaning that the dynamic shear-strain value the specimen can withstand increases.

This is due to changes in the cemented structure of the lightweight soil as EPS particles are added. Additionally, as the number of EPS particles increases, the volume of the soil cavity increases. This increase in the soil’s weak internal surface reduces the force capacity of the cemented structure. This diminishes the ability to withstand deformation and causes a drop in the dynamic shear modulus of the lightweight soil [31]. At the same time, at a small dynamic shear strain (*γ* = 0.0015%), the comparative shift in the particles inside the lightweight soil with EPS particles is small, and it does not cause damage to the specimen. However, as the dynamic shear strain progressively increases, the comparative shift in the particles inside the specimen grows. The material’s microcracks eventually enlarge and connect, generating macroscopic fractures that cause strain softening. As a result, *G* decays more quickly as *γ* increases.

#### 3.2.2. Impact of Confining Pressure on the *G*–*γ* Curve

**Figure 7:**
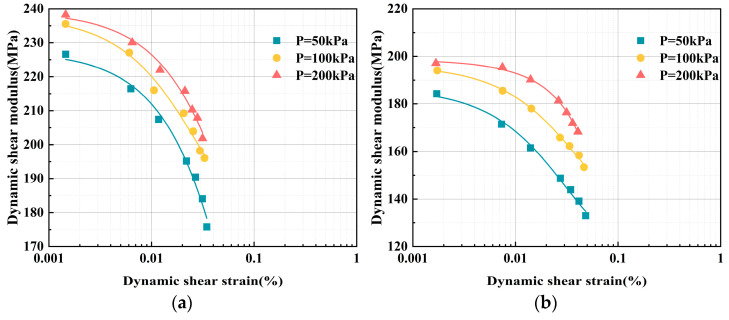

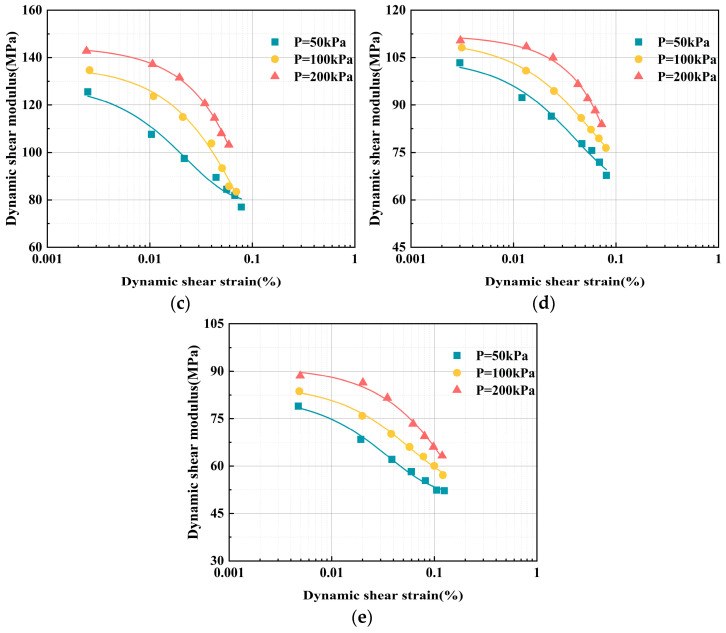
ELS *G*–*γ* relationship curves under different confining pressures. (**a**) EPS particle content = 20%; (**b**) EPS particle content = 30%; (**c**) EPS particle content = 40%; (**d**) EPS particle content = 50%; and (**e**) EPS particle content = 60%.

Since external pressure affects the soil throughout the burial process, in the practical application of ELS, the *G* value changes as the soil pressure increases. To investigate how confining pressure (*P*) influences *G*, the *G*–*γ* relationship curves of ELS under varying confining pressures were obtained for five different EPS particle contents, using the 7-day data as an example, as shown in Figure 7.

This is because, during sample preparation, certain pores develop within the EPS granular lightweight soil’s internal structure. As a result, as *γ* increases, the lightweight soil becomes more porous, and the specimen’s shear strength decreases, which lowers *G*. However, as *P* increases, the frictional force between the EPS particles also increases. This makes it more difficult for the particles to slide and deform under dynamic loading, and, at the same time, it limits the lateral displacement of the lightweight soil, increasing *G*. The EPS particles provide a large amount of compressive deformation space to the specimen [32]. As *P* increases, the specimen becomes compressed and denser, enhancing its resistance to deformation and increasing the dynamic shear modulus. Additionally, the increase in *P* reduces the porosity within the lightweight soil structure. The denser the soil specimen is, the faster the wave propagation speed becomes.

#### 3.2.3. Curing Age Impact on the *G*–*γ* Relationship

**Figure 8:**
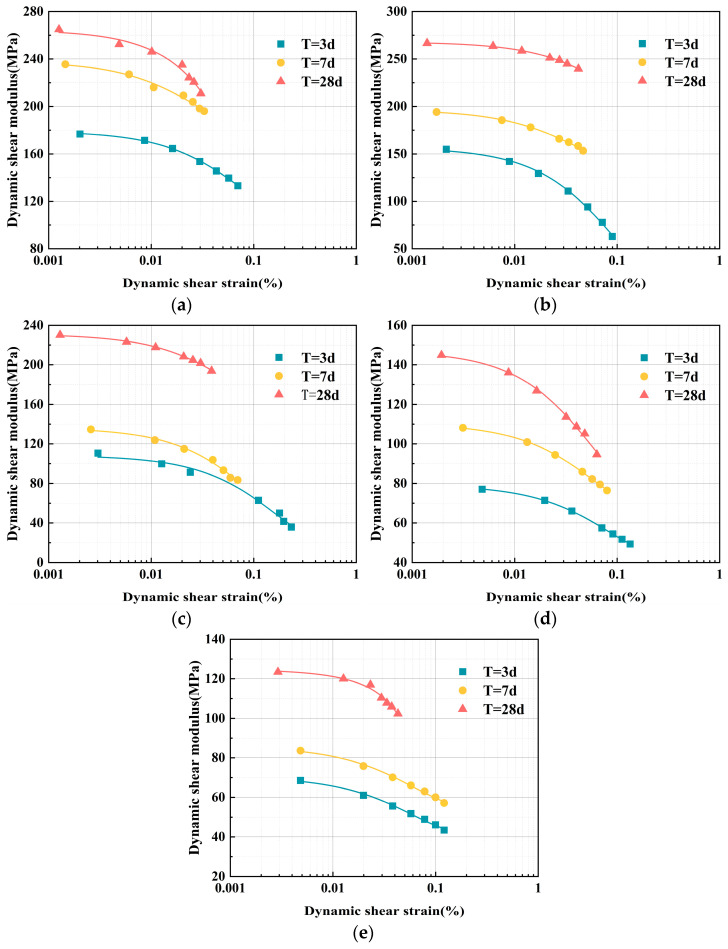
ELS *G*–*γ* relationship curves at different curing ages. (**a**) EPS particle content = 20%; (**b**) EPS particle content = 30%; (**c**) EPS particle content = 40%; (**d**) EPS particle content = 50%; and (**e**) EPS particle content = 60%.

The *G* of ELS varies, and material strength increases as the curing age (T) progresses due to more thorough internal material reactions. To examine the effect of curing age on the *G* of ELS, experimental data at the 100 kPa confining pressure were integrated. The study yielded *G*–*γ* relationship curves for five different EPS particle contents, with curing ages of 3 d, 7 d, and 28 d, as shown in Figure 8.

With a consistent EPS particle content, the *G* of the specimens increases with longer curing ages, peaking at *T* = 28 d. Additionally, the *G* of the specimens shows a nonlinear decrease with an increasing dynamic shear strain at each curing age. At *γ* > 0.01%, the rate of decline accelerates. For example, when the volume mixing ratio of EPS particles reached 30% under the same dynamic shear strain (*γ* = 0.002%), the *G* of the specimens at a 28 d curing age increased by 73 MPa and 112 MPa compared to those at 7 d and 3 d curing ages, respectively. In the range of 0.002% < *γ* < 0.008%, the *G* of the specimens at *T* = 28 d, 7 d, and 3 d decreased by 3%, 7%, and 14%, respectively, compared to the previous strain range. As the dynamic shear strain rises, the rate of reduction accelerates.

This is because the bonding material’s hydration reaction occurs for longer as the curing age of ELS increases, filling the pores with hydration products. This enhances the bonding structure strength of the specimens and increases material stiffness [33]. The hydrophobic nature of the EPS particle surfaces leads to poor adhesion with the bonding material. However, as the curing age increases, hydration products deposit on the EPS particle surfaces, thereby enhancing the bonding capacity between the EPS particles and the bonding material [34]. Simultaneously, EPS particles act as a framework, forming a stable structural framework. With the strengthening of adhesion, the shear resistance of the specimens further improves as the curing age increases [35].

### 3.3. Maximum Dynamic Shear Modulus G_max_

#### 3.3.1. *G*_max_ Computation

The dynamic shear modulus maximum (*G*_max_) is an essential component of soil dynamics; *G*_max_ refers to the maximum stiffness of the soil under shear stress [36]. Since the EPS particles–lightweight soil curve shape follows a hyperbolic form, the Hardin hyperbolic model [37] is used in this study to compute the *G*_max_ of ELS at small strains. The expression for the Hardin hyperbolic model is as follows:

(6) $$\tau =\frac{\gamma }{a+b\cdot \gamma }$$

where *a* and *b* are the fitting parameters, *τ* is the dynamic shear stress (kPa), and *γ* is the dynamic shear strain (%).

Substituting *τ* = *G*·*γ* into Equation (6), the expression for the dynamic shear modulus can be derived, and the linear association between 1/*G* and *γ* can then be revealed, as outlined in Equation (7).

(7) $$\frac{1}{G}=a+b\cdot \gamma$$

**Figure 9:**
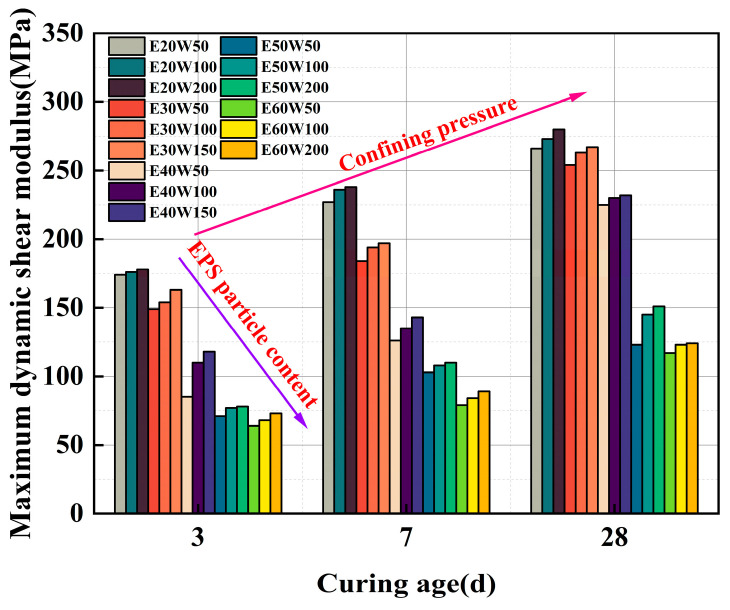
Calculation results of ELS *G*_max_ at different curing ages.

Furthermore, from Equation (7), it is clear that when *γ* approaches 0, 1/*G* = *a*, thereby yielding the maximum dynamic shear modulus *G*_max_ = 1/*a*. Figure 9 presents the calculated results of *G*_max_ under various curing ages. It can be noted that variations in the EPS particle content, restricting pressure, and curing age have different effects on the *G*_max_ calculation.

#### 3.3.2. Impact of the EPS Particle Content on *G*_max_

**Figure 10:**
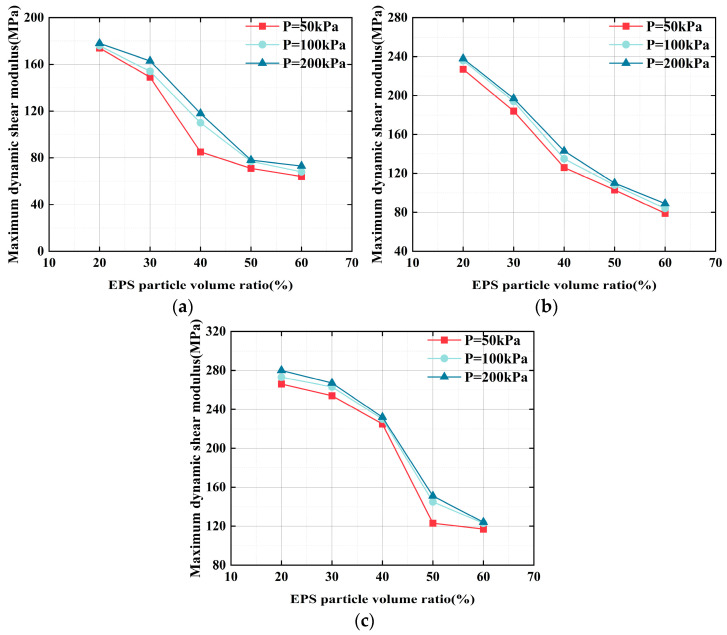
Connection between the EPS particle content and the *G*_max_ of the ELS: (**a**) 3 d curing age; (**b**) 7 d curing age; and (**c**) 28 d curing age.

Figure 10 displays the relationship between the *G*_max_ of the ELS and EPS particle contents. At every confining pressure and curing age, *G*_max_ decreases as the EPS particle content increases. For example, in Figure 10b, *G*_max_ at a curing age of 7 d was 227, 236, and 238 MPa for 50, 100, and 200 kPa confining pressures, respectively. With an increasing EPS particle content, *G*_max_ decreased to 79, 84, and 89 MPa, respectively, representing a relative decrease of 65%, 64%, and 63%. A summary of the *G*_max_ reduction percentage under different EPS contents is shown in Table 3.

#### 3.3.3. Impact of Confining Pressure on *G*_max_

**Figure 11:**
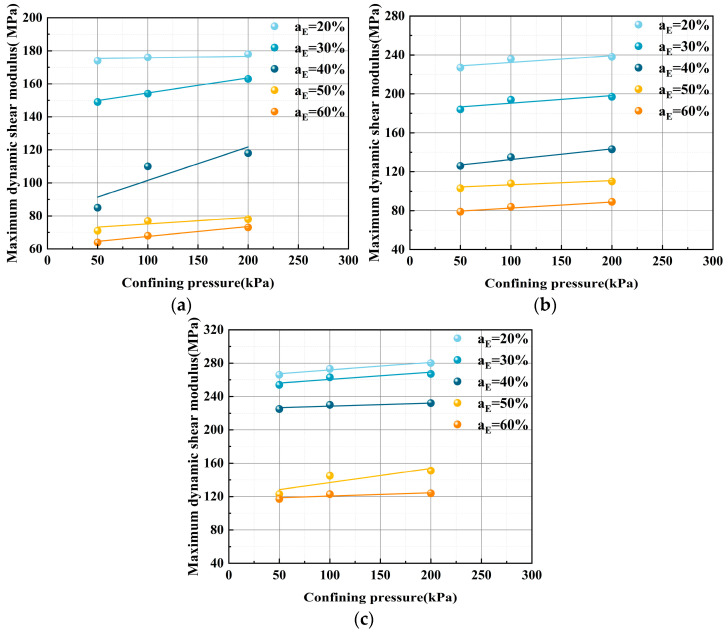
Connection between confining pressure and the *G*_max_ of the ELS: (**a**) 3 d curing age; (**b**) 7 d curing age; and (**c**) 28 d curing age.

Figure 11 demonstrates the relationship between *G*_max_ and the confining pressure of ELS. For each of the five distinct EPS particle contents, the *G*_max_ rises as the restricting pressure increases at the same curing age. For example, the *G*_max_ was 227 MPa, 184 MPa, 126 MPa, 103 MPa, and 79 MPa at a curing age of 7 d. The *G*_max_ increased to 238 MPa, 197 MPa, 143 MPa, 110 MPa, and 89 MPa, respectively, as the confining pressure increased, corresponding to relative increases of 5%, 7%, 14%, 7%, and 12%.

#### 3.3.4. Impact of Curing Age on *G*_max_

**Figure 12:**
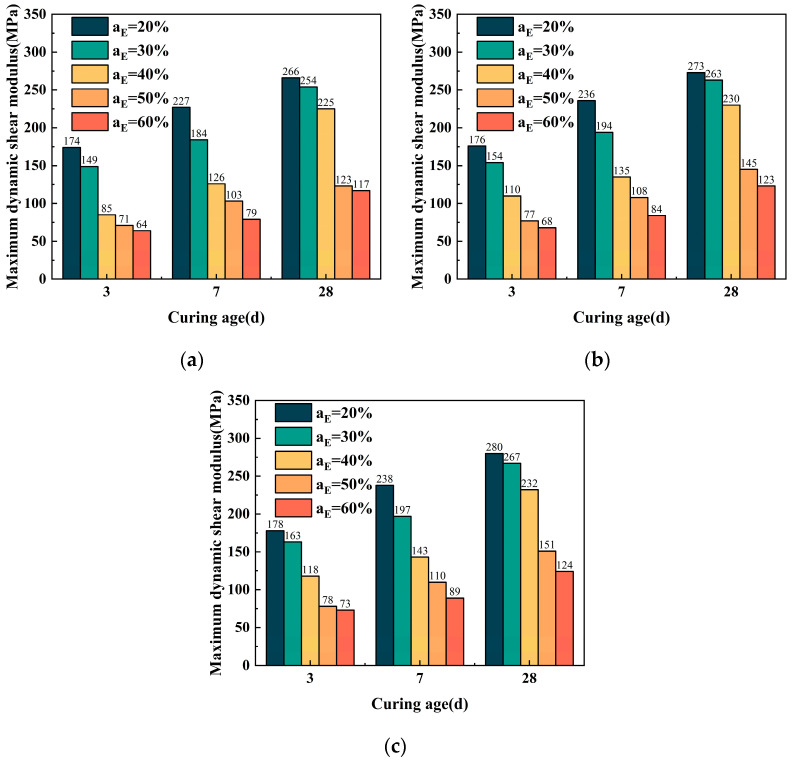
The connection between curing age and the *G*_max_ of the ELS. (**a**) Confining pressure = 50 kPa; (**b**) confining pressure = 100 kPa; and (**c**) confining pressure = 200 kPa.

Figure 12 shows the relationship between the *G*_max_ and curing age. When the curing age increases in similar restricting pressure circumstances, the *G*_max_ with varying EPS particle contents exhibits an increasing trend. For example, at EPS particle contents of 20%, 30%, 40%, 50%, and 60%, the maximum dynamic shear modulus of ELS was 177 MPa, 155 MPa, 111 MPa, 77 MPa, and 69 MPa at 100 kPa, respectively. As the curing age increased, the *G*max increased to 274 MPa, 267 MPa, 230 MPa, 145 MPa, and 123 MPa, with relative increases of 55%, 72%, 108%, 88%, and 80%, respectively. By analyzing the mechanical properties of ELS, students can master the basic skills of resonant column experiments and improve their data processing abilities.

## 4. Discussion

### 4.1. Damage Model

Numerous academics have studied the dynamic constitutive model of lightweight soil. Their findings show that under dynamic loading, soil particles shift and reorganize, breaking down the cement structure of the soil as strain increments, which causes the shear modulus (*G*) to gradually decrease [38,39]. Previous resonance column tests also reveal that the shear modulus (*G*) of ELS decreases with an increased dynamic shear strain, influenced by varying EPS particle contents, confining pressures, and curing ages. This trend supports the development of a small-strain dynamic shear modulus decay model from a damage perspective [40]. Given that the *G*_max_ of ELS remains constant within the small strain range, the decay of *G* with an increasing shear strain (*γ*) is attributed to the internal structural damage of the soil. To create the damage model for *G*, as shown in Equation (8), damage variable *D* is introduced.

(8) $$G=G_{\max}\left(1-D\right)$$

Equation (8) outlines the attenuation of the minor strain *G* with increasing *γ*, reflecting its clear physical significance [41]. From Equation (8), it can be seen that *G* decreases as *D* increases, with *D* gradually rising alongside *γ*. Theoretically, *D* = 0 when the soil is intact, and *D* = 1 when *γ* increases to a sufficiently large value. However, in reality, due to the range of the small-strain control, *D* < 1 [42].

The analysis reveals a close relationship between *D* and *γ*. Research by Yan [43] and others has shown that *D*’s damage evolution is well described by the Weibull distribution function. Therefore, as shown in Equation (8), according to reference [42], the Weibull distribution function is added to simulate the decay process of *G*.

(9) $$D=1-\exp\left[-{\left(\frac{\gamma }{\gamma_{0}}\right)}^{m_{0}}\right]$$

where *γ*_0_ and *m*_0_ are parameters of the Weibull distribution function.

Finally, by combining Equation (9) with Equation (8), we obtain Equation (10). This equation represents the damage model for the attenuation of *G* in ELS.

(10) $$G=G_{\max}\exp\left[-{\left(\frac{\gamma }{\gamma_{0}}\right)}^{m_{0}}\right]$$

**Figure 13:**
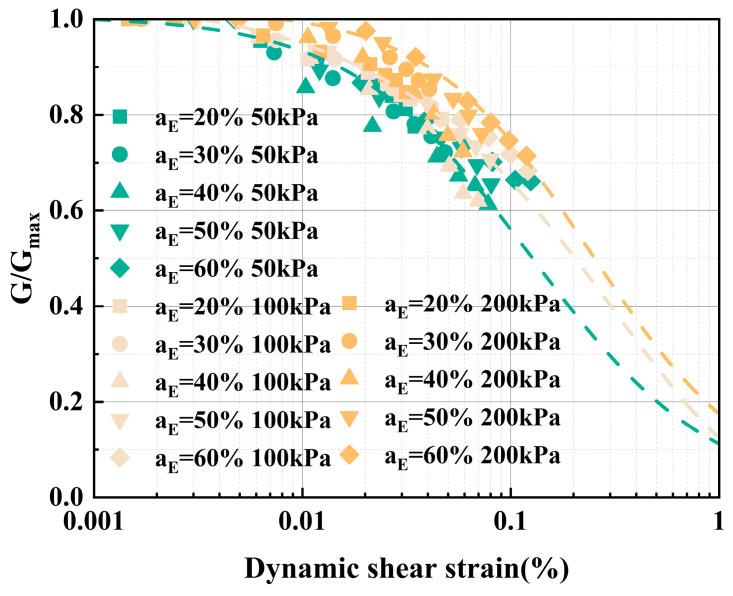
Normalized decay curves of *G*.

The normalization of *G* provides a better reflection of its decay with increasing *γ* [44]. Based on resonance column test data, *G* values for different EPS contents (*a*_E_) and confining pressures (*P*) are normalized as *G*/*G*_max_, and the decay curves of *G*/*G*_max_ are plotted, as shown in Figure 13. Under the same conditions, the confining pressure and EPS particle content typically have little effect on *G*/*G*_max_, exhibiting a pattern of gradual initial decay followed by a rapid decline overall. However, *G*/*G*_max_ under higher confining pressures shows a gentler overall change compared to lower pressures. From a damage perspective, higher confining pressure constrains particle displacement within the soil mass, reducing particle movement and thereby slowing down damage to the ELS structure, resulting in a decrease in the magnitude of *G*/*G*_max_. The Hardin–Drnevich model, Ramberg–Osgood model, and Maitin–Davidenkov model are commonly used to describe the constitutive relationship of small-strain dynamic characteristics in soil, the specific form and application range are shown in Table 4. Among these models, the Hardin–Drnevich model has a relatively simple parameter selection in its formula, which reduces complexity [45]. This model is applicable to various soil types, especially in small strain ranges, and has good fitting effects. In resonance column tests, the strain range of the test is usually between 0.001% and 0.1%, and the Hardin–Drnevich model can precisely meet the testing requirements of the resonance column test. Meanwhile, within the small strain range, the dynamic shear modulus of the soil decreases with increasing dynamic shear strain. The Hardin–Drnevich model can effectively describe the attenuation behavior of the soil dynamic shear modulus and express it through a simple formula [46]. The Ramberg–Osgood model is commonly used for high-precision fitting requirements and is more suitable for large strain ranges. The Maitin–Davidenkov model has complex parameter selection, and both models have limitations in their applicability to soil types [47,48,49]. Therefore, the Hardin–Drnevich (H-D) model is employed to explain the attenuation process of *G*.

### 4.2. Model Comparison

The H-D model reflects the attenuation of the soil’s *G* under small strains [50]. This research fits the trial data for both the H-D model and the damage model to examine the differences between the two models and confirm the precision of the damage model.

The expression for the H-D model is shown in Equation (11):

(11) G=Gmax1+γγr

where *γ_r_* is the reference shear strain (%).

Model parameters for both the H-D model and the damage model were derived through fitting calculations and are presented in Table 3.

**Figure 14:**
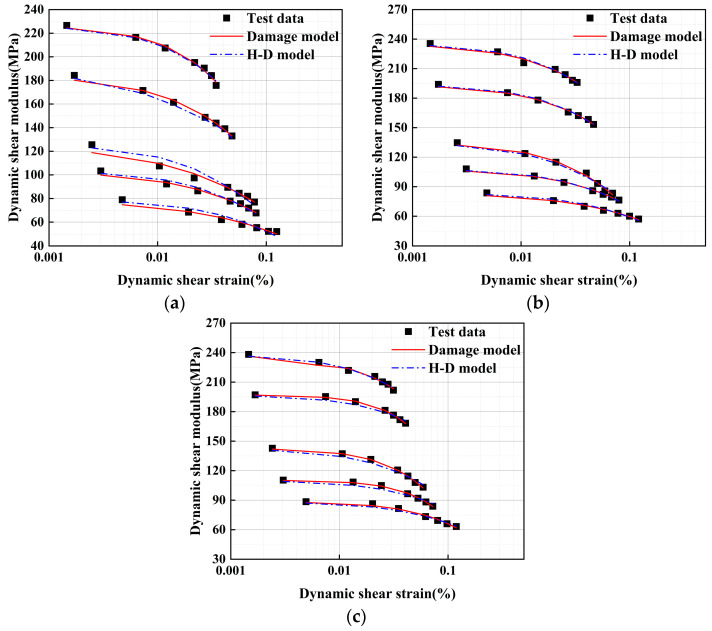
Comparison of experimental data and model results. (**a**) Confining pressure = 50 kPa; (**b**) confining pressure = 100 kPa; and (**c**) confining pressure = 200 kPa.

The experimental data were fitted using Equations (10) and (11), and the fitting results are shown in Figure 14. It demonstrates that the damage model fitting curves of lightweight soil with different EPS particle contents under varying confining pressures exhibit certain similarities with the fitting curves of the H-D model and the experimental data obtained from the resonant column tests. To compare the accuracy of the two models more intuitively, the coefficient of determination *R*^2^ was calculated:

(12) $$R^{2}=1-\frac{\sum_{i=1}^{n}{\left(x_{i}-x\right)}^{2}}{\sum_{i=1}^{n}\left(x_{i}-{\bar{x}}_{i}\right)}$$

where *x_i_* is the dynamic shear modulus measured from the resonant column test (MPa); ${\bar{x}}_{i}$ is the average value of the dynamic shear modulus measured from the tests (MPa); and *x* is the dynamic shear modulus obtained from the model calculations (MPa). The closer the coefficient of determination *R*^2^ is to one, the higher the accuracy of the model fitting.

**Figure 15:**
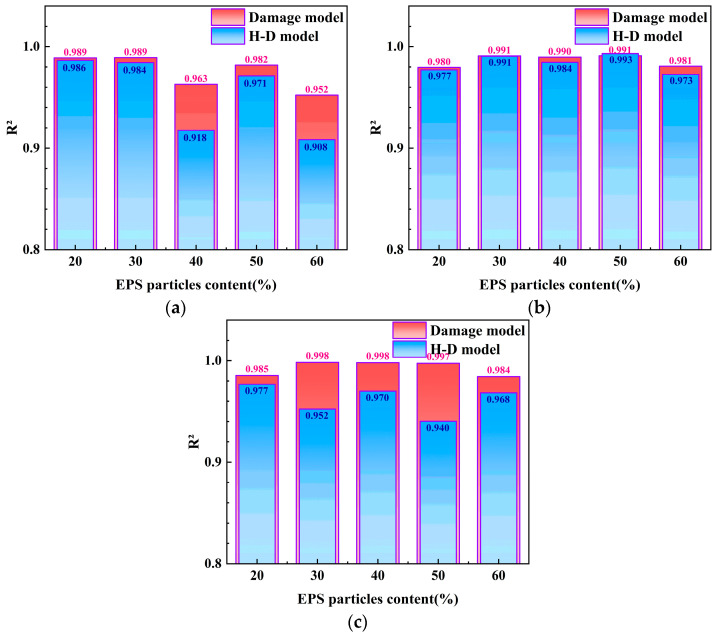
Comparison of the R^2^ values of the damage model and the H-D model. (**a**) Confining pressure = 50 kPa; (**b**) confining pressure = 100 kPa; and (**c**) confining pressure = 200 kPa.

Figure 15 shows the coefficient of determination (*R*^2^) values for both the damage model and the H-D model. By comparing the *R*^2^ values, it was found that the fitting accuracy of the damage model is generally better than that of the H-D model, especially under a confining pressure of 200 kPa, where the *R*^2^ of the damage model is mostly above 0.98, while the *R*^2^ of the H-D model ranges from 0.94 to 0.97. Therefore, it can be concluded that the damage model better reflects the attenuation law and damage process of the dynamic shear modulus of EPS particles in lightweight soil under small strains compared to the H-D model. Meanwhile, to validate the applicability of the damage model to other materials, the resonant column test data of cement-stabilized silty clay and cement–fly ash-stabilized silty clay under a confining pressure of 50 kPa, as reported by Lang et al. [19], were introduced. The damage model described by Equation (10) demonstrates a certain degree of applicability in characterizing the attenuation law of the dynamic shear modulus at small strains for other materials.

### 4.3. Analysis of Damage Model Parameters

**Figure 16:**
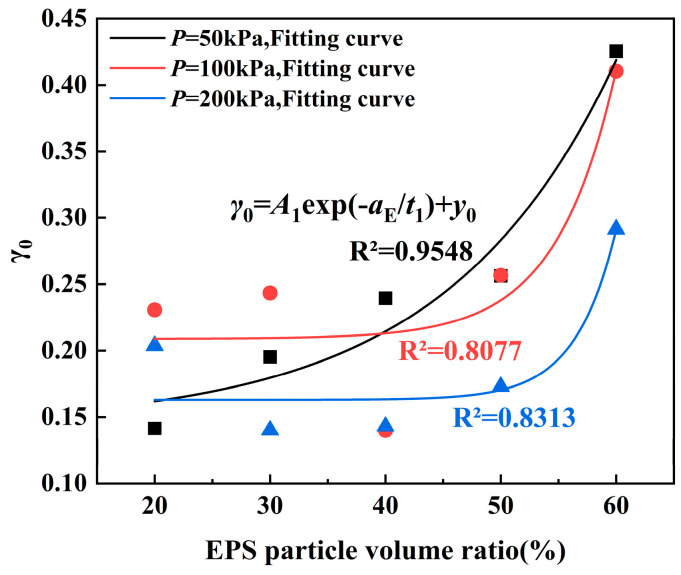
Connection between *γ*_0_ and the EPS particle content.

**Figure 17:**
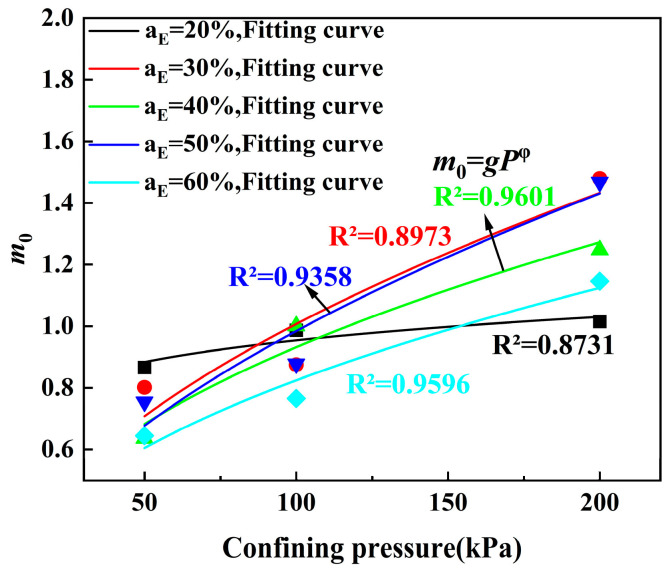
Relationship between confining pressure and *m*.

The conclusion in Section 4.2 is that the damage model has a better effect on characterizing the attenuation of the ELS shear modulus under a small strain. As shown in Table 5, 15 groups of different damage parameters (*γ*_0_ and *m*_0_) can be obtained under different confining pressures and EPS particle contents. Since the EPS particle content and confining pressure have different influences on the damage model parameters *γ*_0_ and *m*_0_ in Equation (10), in order to better explore the change rule of *γ*_0_ and *m*_0_, the relationships between the damage parameter *γ*_0_ and EPS particle content and between m and confining pressure are established, as shown in Figure 16 and Figure 17. As can be seen from Figure 16, with the increase in the EPS particle content, parameter a generally shows an increasing trend. The relationship between parameter a and the EPS particle content is expressed in Equation (13):

(13) $$\gamma_{0}=A_{1}\exp\left(\frac{-a_{E}}{t_{1}}\right)+y_{0}$$

where *A*_1_, *t*_1_, and *y*_0_ are the model parameters and a_E_ is the EPS particle content (%).

Figure 17 shows that as the confining pressure increases, *m*_0_ also shows an increasing trend. Their functional relationship is expressed by Equation (14):

(14) $$m_{0}=g\cdot P^{\varphi }$$

where *g* and *φ* are model parameters and *P* is the confining pressure (kPa).

By substituting Equation (13) and Equation (14) into Equation (10), the damage model considering the EPS particle content and confining pressure can be obtained. In road engineering design, EPS lightweight mixtures are commonly used to improve the soil-bearing capacity and reduce structural load, with the dynamic shear modulus being a crucial parameter. This damage model can predict the dynamic shear modulus of ELS under small strain ranges for different EPS particle contents and confining pressures, providing a theoretical basis for road construction engineering. At the same time, establishing models and comparing them can improve students’ innovation ability. Table 6 shows the advantages and disadvantages of ELS in this study.

### 4.4. Effectiveness of Teaching Reform

In the teaching of engineering detection experiments, the main purpose is to achieve two objectives. Objective 1 involves using modern testing tools to test the material’s mechanical properties and structural load-bearing properties and obtain effective testing data to establish engineering awareness. Objective 2 is able to apply theoretical and experimental research to analyze detection data, obtain reasonable conclusions, and improve innovation capabilities. According to Equation (15), the achievement degree of each teaching objective (*ACO_i_*) can be calculated [20]:

(15) $${ACO}_{i}=\frac{\sum_{j=1}^{n}\left(S_{i}^{j}\times a_{j}\right)}{100\times \sum_{j=1}^{n}a_{j}b_{i}^{j}}$$

where $S_{i}^{j}$ represents the average score of the *j*th assessment method for the *i*th teaching objective on a percentage scale, *a_j_* represents the proportion of the *j*th assessment method, $b_{i}^{j}$ represents the support weight of the *j*th assessment method for the *i*th teaching objective, and *n* represents the total number of assessment methods.

**Figure 18:**
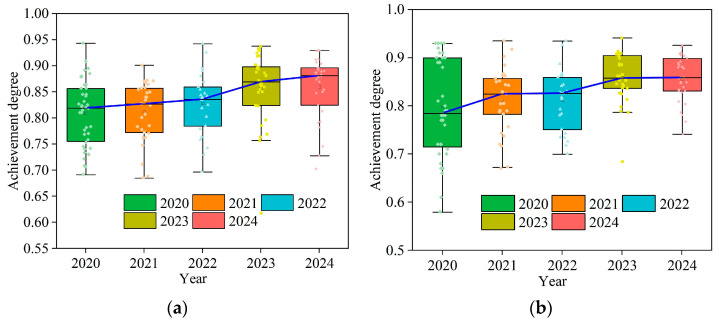
Calculation results of achievement degree of teaching objectives. (**a**) Achievement degree of teaching objective 1; (**b**) achievement degree of teaching objective 2.

This study used a resonance column test to obtain the dynamic performance of ELS under small strain conditions, and a damage model was established to illustrate the attenuation process of *G* with a shear strain (*γ*) of ELS through data analysis. The reform of the testing materials, testing methods, and data processing methods in experimental teaching has fulfilled the teaching goals, and the achievement degree calculation results are shown in Figure 18. The implementation of the experiment teaching reform began in 2020, and the achievement of the teaching objectives over the five years from 2020 to 2024 was higher than 0.7 and increased year by year. It can be seen that diverse experimental materials, advanced testing techniques, and data processing methods can effectively improve students’ learning and innovation abilities.

## 5. Conclusions

In roadbed filling engineering, EPS particle lightweight soil, as a lightweight filler, possesses the characteristics of low density and high strength, which can effectively reduce the mass of filling materials, thereby stabilizing slopes and mitigating the uneven settlement of soft soil roadbeds. This study employs resonant column tests to investigate the influence of the EPS particle content, confining pressure, and curing age on the small-strain shear modulus (*G*) of ELS and establishes a damage model to illustrate the attenuation process of *G* with shear strain (*γ*). The research results provide theoretical guidance for the application of ELS in soft soil roadbed engineering. The main conclusions are as follows:

(1) Increasing the EPS particle content reduces the *G* of ELS. Due to the decreased load-bearing capacity of the internal structure, there is greater relative displacement between the EPS particles. With curing ages of 3, 7, and 28 days and varying confining pressures, *G*_max_ (ranging from 64 MPa to 280 MPa) decreased by 61%, 64%, and 55%, respectively, as the EPS particle content increased. Additionally, the *G* of ELS decreased with increasing *γ* across different EPS particle contents.
(2) Elevating confining pressure boosts the *G* of ELS. Greater confining pressure restricts the lateral displacement of the soil mass, resulting in higher specimen compression and an increase in *G* with rising confining pressure. At 3, 7, and 28 days of curing, *G*_max_ increased by 15%, 9%, and 8%, respectively, with increasing confining pressure. Furthermore, the *G* of ELS decreased with increasing *γ* across various confining pressures.
(3) Extended curing ages enhance the *G* of ELS. As curing ages increase, the hydration reactions of the binding material become more thorough, resulting in an increase in *G* with longer curing periods. Under confining pressures of 50, 100, and 200 kPa, *G*_max_ increased by 53%, 55%, and 57%, respectively, with extended curing ages. Likewise, the *G* of ELS decreased with increasing *γ* under different curing ages.
(4) The reduction in *G* of ELS with increasing *γ* at small strains is fundamentally due to the lightweight soil’s structural deterioration. The damage model, incorporating the damage variable *D*, effectively reflects the attenuation pattern and damage process of the *G* of ELS at small strains. The parameter *γ*_0_ and EPS particle content are functionally related, with *γ*_0_ ranging from 0.14 to 0.43. The parameter *m*_0_ and confining pressure are also functionally related, with *m*_0_ ranging from 0.64 to 1.48.

This study investigated the dynamic shear modulus of ELS within the small strain range of 10^−6^ to 10^−4^ through resonant column tests, considering the effects of different EPS particle contents, confining pressures, and curing ages on the dynamic shear modulus. ELS, a low-density geosynthetic material, effectively reduces building material mass, stabilizes slopes, and alleviates the subgrade settlement of soft soil. It exhibits a high dynamic shear modulus under small strains (10^−6^–10^−4^), which improves with increased confining pressure and curing age. However, a higher EPS particle content significantly reduces its dynamic and maximum shear modulus, leading to mechanical property deterioration and structural degradation under shear strain. Additionally, a damage model related to the EPS particle content and confining pressure was established, which effectively describes the attenuation pattern of the dynamic shear modulus of ELS under small strains. However, due to the limitations of the resonant column apparatus, all tested samples were small-sized cylindrical specimens. During the testing process, the boundary effects of the specimens may cause deviations from ideal conditions, potentially influencing the test results. Moreover, the small size of the specimens may not accurately represent the actual dynamic characteristics of the soil.

This experimental study is part of an engineering detection laboratory course. In contrast to the original experimental program, which focused on the mechanical properties of geotechnical materials, this study utilized the resonant column testing system to examine the dynamic properties of EPS lightweight soil. Additionally, a shear model prediction model of ELS was developed. This research introduces innovations in experimental materials, testing methods, and data analysis, offering opportunities to enhance students’ practical skills, broaden their knowledge, and foster their ability to innovate. Furthermore, the detection experiment of this project covers multiple aspects, such as material preparation, performance testing, and experimental result analysis, involving multiple disciplines, such as environmental science, material science, and soil mechanics. In terms of engineering education and methods, it is an effective exploration of the reform of civil engineering experimental teaching and has achieved positive teaching results. Through this experiment, students can not only stimulate their interest in interdisciplinary research but can also significantly improve their engineering experimental skills and innovation abilities. This experiment combines theory with practice in civil engineering experimental teaching, laying a solid foundation for the cultivation of comprehensive applied talents in universities.

## Figures and Tables

**Table 1:** Physical and mechanical characteristics of soil.

Plastic Limit (%)	Liquid Limit (%)	Plasticity Index	Liquidity Index	Natural Water Content (%)	Specific Gravity
23	37	14	0.43	29	2.88

**Table 2:** Resonance column test program.

EPS Particle Content (%)	Cement (%)	Sodium Silicate (%)	Water Content (%)	Curing Age (d)	Confining Pressure (kPa)
20, 30, 40, 50, 60	20	6	90	3, 7, 28	50, 100, 200

**Table 3:** Percentage reduction in *G*_max_ under different EPS particle contents.

Curing Age (d)	Confining Pressure (kPa)	EPS Content (%)	*G*_max_ (MPa)	*G*_max_ Decline Percentage (%)
			64–178	64
3–28	50–200	20–60	79–238	67
			117–280	58

**Table 4:** Small-strain dynamic constitutive model of soil mass.

Model	Expression	Strain Range	Applicable Type	Reference Source
Hardin–Drnevich model	G=Gmax1+γγr	0.001–1%	Various soil types	Fu [45] Hou [46]
Ramberg–Osgood model	$G_{\max}=\frac{\tau }{\gamma }\left(1+{\left(\frac{\tau }{A_{1}}\right)}^{n-1}\right)$	0.01–5%	Often used on clay and fine-grain soils	Primusz [47] Ahn [48]
Maitin–Davidenkov model	$G=G_{\max}\left(1-u\gamma^{v}\right)$	0.001–10%	Complex soil types	Wu [49]

**Table 5:** Model parameter computation results.

Confining Pressure	EPS Particles Volume Ratio (%)	Damage Model		*R* ^2^	H-D Model	*R* ^2^
		*γ*_0_ (%)	*m*_0_		*γ_r_* (%)	
	20	0.14139	0.86659	0.98896	0.13142	0.98649
	30	0.19530	0.80207	0.98922	0.12134	0.98392
50 kPa	40	0.23946	0.63587	0.96294	0.11212	0.91764
	50	0.25624	0.75463	0.98174	0.14869	0.97121
	60	0.42529	0.64484	0.95232	0.19515	0.90845
	20	0.23067	0.98693	0.97956	0.15903	0.97718
	30	0.24339	0.87549	0.99093	0.17341	0.99059
100 kPa	40	0.13985	1.00101	0.98968	0.11535	0.98450
	50	0.25658	0.87810	0.99108	0.18476	0.99317
	60	0.41034	0.76591	0.98066	0.23946	0.97266
	20	0.20365	1.01547	0.98588	0.18551	0.98536
	30	0.14004	1.47926	0.99830	0.26403	0.95235
200 kPa	40	0.14290	1.24764	0.99807	0.16894	0.96993
	50	0.17282	1.46766	0.99745	0.26495	0.94028
	60	0.29132	1.14622	0.98428	0.30510	0.96832

**Table 6:** Advantages and disadvantages of FSCS.

Advantage	① Low density; lightweight; can ease the uneven subgrade settlement of soft soil. ② In the small strain range (10^−6^–10^−4^), it has a high dynamic shear modulus; it can also resist shear deformation. ③ Increasing confining pressure and prolonging curing age can significantly increase dynamic shear modulus and enhance mechanical properties. ④ Combining material science, soil mechanics, and environmental protection principles, it has environmental protection significance.
Disadvantage	① With the increase in the EPS particle content, the dynamic shear modulus (*G*) and maximum shear modulus (*G*_max_) decreased significantly, and the mechanical properties decreased. ② In the small strain range, the dynamic shear modulus (*G*) decreases with the increase in shear strain (*γ*), indicating that the structure will gradually degenerate during the stress process.

## Data Availability

Data are contained within the article.

## Glossary

Abbreviation	Implication
EPS	Expanded polystyrene
ELS	Expanded polystyrene particles in lightweight soil
H-D	Hardin–Drnevich model
*G*	Dynamic shear modulus (MPa)
*G*_max_	Maximum dynamic shear modulus (MPa)
*ACO*_i_	Achievement degree of each teaching objective
Variables	Implication
*P*	Confining pressure (kPa)
*I*	Moment of inertia of the sample (kg·m^2^)
*m*	Mass (kg)
*d*	Outside diameter (m)
*I*_0_	Moment of inertia of the resonant column system (kg·m^2^)
*V*_s_	Shear wave velocity (m/s)
*f*	Resonance frequency obtained from the resonant column test (Hz)
*H*	Specimen’s height (m)
*β*	Torsional vibration frequency equation’s eigenvalue
*ρ*	Density of the specimen (t/m^3^)
*a*_E_	EPS particle content (%)
*γ*	Dynamic shear strain (%)
*G*-*γ*	Dynamic shear modulus and dynamic shear-strain relationship
*a*,*b*	Empirical constants
*τ*	Dynamic shear stress (kPa)
*D*	Damage variable
*γ*_0_,*m*_0_	Parameters of Weibull distribution function
*γ_r_*	Reference shear strain (%)
*R* ^2^	Correlation coefficient
*x_i_*	Dynamic shear modulus measured from the resonant column test (MPa)
${\bar{x}}_{i}$	Average value of the dynamic shear modulus measured from the test (MPa)
*x*	Dynamic shear modulus obtained from the model calculations (MPa)
*A*_1_, *t*_1_, *y*_0_, *g*, *φ*	Model parameters
*T*	Curing age (d)
*V*	Resonance output voltage (Volts)
$S_{i}^{j}$	Average score of the *j*th assessment method for the *i*th teaching objective on a percentage scale
*a*_j_	Proportion of the *j*th assessment method
$b_{i}^{j}$	Support weight of the *j*th assessment method for the *i*th teaching objective.
*n*	Total number of assessment methods
